# SOCIAL ROLE VALORIZATION IN COMMUNITY MENTAL HEALTH HOUSING: DOES IT CONTRIBUTE TO THE COMMUNITY INTEGRATION AND LIFE SATISFACTION OF PEOPLE WITH PSYCHIATRIC DISABILITIES?

**DOI:** 10.1002/jcop.21524

**Published:** 2013-02-14

**Authors:** Tim Aubry, Robert J Flynn, Barb Virley, Jaclynne Neri

**Affiliations:** University of Ottawa

## Abstract

Despite its importance as a theory in the development of programs for populations with disabilities, social role valorization (SRV) has received relatively little attention in community mental health research. We present findings of a study that examined the relationship of housing-related SRV to community integration and global life satisfaction of persons with psychiatric disabilities. The housing environments and associated supports of a group of 73 persons with psychiatric disabilities living in a mid-sized city were assessed using the PASSING rating system on the extent that their housing environments facilitated SRV. In addition, in-person interviews were conducted to determine the levels of physical integration, psychological integration, social integration, and life satisfaction of study participants. Results showed SRV contributing directly to all three types of community integration. Psychological integration was found to mediate the relationship between SRV and life satisfaction. Implications of the findings are discussed. © 2013 Wiley Periodicals, Inc.

Social role valorization (SRV) is defined as the use of culturally valued means to enable, establish, enhance, maintain, and/or defend valued social roles for people at value risk (Wolfensberger, 1985, 1998, 2000). The overall of goal of SRV is to create social roles for devalued populations that enhance their image and personal competencies. SRV as a theory was preceded by and evolved from the concept of normalization. Normalization is defined as “the utilization of means which are as culturally normative as possible in order to establish and/or maintain personal behaviours and characteristics which are as culturally normative as possible” (Wolfensberger, 1972, p. 28). Normalization was originally formulated within the context of the deinstitutionalization of people with developmental disabilities and it focused on providing these individuals with a life in “normalized” settings in the community (Nirje, 1969).

In follow-up to normalization, Wolfensberger (1985) posited that people with disabilities needed SRV or valued social participation within the community. This new conceptualization highlighted the critical importance of improving public perceptions of devalued persons in society by supporting them to achieve physical and social integration outside of treatment settings and in the community (Wolfensberger, 1983, 1992, 2000). Opportunities for SRV in which devalued individuals adopt valued roles in the context of relationships with non-devalued individuals are expected to produce benefits such as life satisfaction, self-esteem, and personal competencies (Wolfensberger & Thomas, 1981). The primary mechanisms for creating these valued social roles are social programs in such areas as housing, employment, and education.

To assist service providers with implementing programs that are based on normalization, the evaluation and teaching tool called “Program Analysis of Service Systems: A Method for the Quantitative Evaluation of Human Services” (PASS; Wolfensberger & Glenn, 1975) was developed as well as a revised version integrating SRV principles known as “Program Analysis of Service Systems’ Implementation of Normalization Goals” (PASSING; Wolfensberger & Thomas, 1983). Essentially, these tools enable an assessment of the extent that programs and service settings are in line with normalization and SRV principles.

Flynn (1999) noted that both PASS and PASSING have been used mostly for pedagogical and program improvement purposes. The results of evaluations of a large number of programs for people with different disabilities around the world using PASS or PASSING can be summarized in the following manner: (a) service quality of programs scores relatively low with regard to normalization and SRV standards; (b) community-based programs that offer some form of housing score higher on the two tools than institutional settings; and (c) programs are rated much higher on their structural characteristics (i.e., location and the extent they facilitate accessibility) than on their functional characteristics (i.e., how they deliver services to their participants) (Flynn, Guirguis, Wolfensberger, & Cocks, 1999). A small number of studies in the area of developmental disabilities have shown a positive relationship between the degree of SRV in program settings and outcomes such as adaptive functioning, community integration, and quality of life of program participants (Borthwick-Duffy, Widaman, Little, & Eyman, 1992; Burchard, 1999; Heal & Daniels, 1986; Hull & Thompson, 1980; Perry & Felce, 1995; Picard, 1988).

Normalization and social role valorization are credited with shifting social policy in many parts of the world towards the social integration of marginalized populations, especially in the field of developmental disabilities (Kendrick, 1999). Undoubtedly, the evolution of community mental health services over the past four decades has also been informed by normalization and SRV but its influence as an organizing theory for mental health systems and services has been largely indirect in nature (Flynn & Aubry, 1999). As a philosophy of service delivery, SRV holds many similar values as those of “recovery-oriented services” that are now being promoted in mental health systems across North America (Davidson & White, 2007). Notably, in line with the tenets of SRV, recovery-oriented services are intended to facilitate consumer's empowerment and independence, opportunities for assuming normal valued roles, the development of social networks that include nondisabled persons and nonprofessionals, and the integration into the community as citizens (Davidson et al., 2007; Farkas, Gagne, Anthony, & Chamberlain, 2005; Jacobson & Curtis, 2000; Sowers, 2005).

Evidence of the indirect role that normalization and SRV has played in the development of community mental health services is apparent in the relatively small amount of research conducted in the area that uses SRV as a guiding theory. In fact, our review of the extant literature identified only four published studies that have examined the degree that normalization and SRV are present in program environments of people with psychiatric disabilities. All of these studies used the PASS instrument.

In Great Britain, Carson, Dowling, Luyombya, Senapati-Sharm, and Glynn (1992) used PASS to compare two in-patient psychiatric units with a residential program based on normalization principles and delivered on the same hospital grounds as the in-patient units. The researchers also assessed patient behavior, quality of life, resident satisfaction, and staff attitudes to treatment and ward management. Carson and his colleagues found that compared to inpatients, the clients in the residential program had higher levels of quality of life, were exposed to more individualized and resident-centered management practices, and were more often accompanied into the community by staff.

In the largest study on normalization conducted to date in mental health, Hull and Thompson (1981a) used an adapted version of PASS to investigate the extent individual, residential, and community characteristics were associated with the adaptive functioning of former psychiatric hospital patients living in board and care homes in Manitoba. Findings showed that the presence of higher levels of different aspects of normalization in housing environments as measured by PASS were predictive of higher levels of different dimensions of adaptive functioning. Overall, Hull and Thompson found that residential variables measured by PASS accounted for more of the variance of adaptive functioning than did the individual characteristics of residents.

Based on the same study, Hull and Thompson (1981b) also reported on findings of determinants of level of normalization in a residence as measured by PASS. Their results showed that residences demonstrating higher levels of normalization were smaller, provided more opportunities to its residents for independence, served only one disability group, and were located in middle-income neighborhoods facilitating access to community resources and social integration opportunities.

In the only other published study, Hull, Keats, and Thompson (1984) examined the environmental quality in community residences for adults with psychiatric disabilities and adults with developmental disabilities and the adaptive functioning of tenants in these residences in Manitoba. PASS was used to measure the level of normalization afforded by the residential environment. In line with the findings of the other studies, a higher level of normalization present in a residence was related to greater adaptive functioning for both tenants with psychiatric disabilities and tenants with developmental disabilities.

Similar to findings in the field of developmental disabilities, the small amount of empirical research conducted to date in community mental health has demonstrated that residential environments promoting normalization and social role valorization are associated with better outcomes in achieving adaptive functioning. Although only examined in a limited and largely indirect manner in the normalization and SRV studies in community mental health, “community integration” (i.e., achieving meaningful participation in the community) for people with psychiatric disabilities has been a central goal of community mental health services since the initiation of deinstitutionalization (Carling, 1995; Segal & Aviram, 1978).

Segal and Aviram (1978) conducted the first study on community integration of persons with psychiatric disabilities in which they examined the “social integration” of residents with psychiatric disabilities who were living in sheltered care facilities in California. Much of the subsequent research on community integration in the two decades following this landmark study relied on the original conceptualization of what Segal and Aviram termed “external social integration,” which they defined as being present, accessing services, and participating in activities with other people in the community (Flynn & Aubry, 1999).

Research has shown that housing characteristics are important predictors of external social integration. In particular, housing characteristics predictive of greater external social integration have included environments that facilitated more tenant involvement, support, spontaneity, and autonomy and communicated clear expectations of residents, provided training opportunities in practical skills, and promoted contact with families and neighbors (Segal & Aviram, 1978). Other housing characteristics related to greater social integration included staff practices individualized to residents’ needs and focused on social skills training and congregate housing, as opposed to nursing or board and care homes (Kruzich, 1985; Nelson, Hall, Squire, & Walsh-Bowers, 1992). Overall, research in this area suggests that housing characteristics in line with principles associated with normalization and SRV contribute to external social integration.

Building on the seminal work of Segal and Aviram (1978), a broader multidimensional definition of community integration has been proposed in the literature that includes physical, social, and psychological dimensions of integration (Aubry & Myner, 1996; Wong & Solomon, 2002). In line with external integration as conceptualized by Segal and Aviram (1978), Aubry and Myner (1996) defined *physical integration* as the extent to which an individual initiates and participates in activities and uses services outside his or her home. They also defined *social integration* as involving the extent that an individual interacts with his or her neighbors (Unger & Wandersman, 1985). Finally, Aubry and Myner defined *psychological integration* as having a sense of community with neighbors that includes perceiving oneself as being a member of the community, having an emotional connection with neighbors, believing in his or her ability to have needs met through neighbors, and having influence in the community (McMillan & Chavis, 1986).

In comparing the community integration of persons with psychiatric disabilities and their neighbors, Aubry and Myner (1996) found that people with psychiatric disabilities living in board and care homes and congregate housing reported similar levels of physical integration and psychological integration as their neighbors. In contrast, people with psychiatric disabilities in the study indicated having lower levels of social integration in comparison to their neighbors. People with psychiatric disabilities had only very limited contact with neighbors beyond saying hello or talking to them on the street. Of the three types of community integration only psychological integration was found in the study to have a significant correlation with general life satisfaction.

More recently, Yanos, Stefanic, and Tsemberis (2011, 2012) compared the community integration of tenants of supported housing programs to a group of community residents living in the same neighborhoods in the Bronx in New York City. Their results showed tenants reporting similar levels of psychological integration (Yanos et al., 2011) but lower levels of physical integration (participation in local activities external to their home), social integration (interaction with neighbors), and citizenship (involvement in political activities and volunteering) than community residents (Yanos et al., 2012). Among tenants of supported housing, greater neighborhood disadvantage, lower immigrant concentration, and lower levels of perceived neighborhood quality predicted lower levels of psychological integration for mental health consumers (Yanos et al., 2011). As well, more severe psychiatric symptoms, higher levels of depression, higher levels of education, and having lived longer in one's current residence were associated with higher levels of social integration.

In other published research focusing on predictors of community integration, Yanos, Felton, Tsemberis, and Frye (2007) found that formerly homeless individuals with severe mental illness living in independent housing who engaged in meaningful activity external to their housing had a greater sense of community within their neighborhood and more social contact with their neighbors. The results were interpreted as showing how the combination of independence and opportunity to make choices associated with living in their own housing facilitated community integration. In another study of adults with psychiatric disabilities who were receiving either supported housing in scattered site apartments or standard care, Gulcur, Tsemberis, Stefanic, and Greenwood (2007) reported that having choice on where to live and living in supported housing predicted both psychological integration and social integration.

Finally, Tsai, Mares, and Rosenheck (2012) examined the extent that social integration improved over the course of a period of 1 year for tenants of supported housing. Although tenants experienced significant improvements in housing stability over the year, they showed only small increases in participation in community activities and civic engagement and no changes in the social support they received. Tsai and his colleagues interpreted their findings as showing tenants to be socially isolated 1 year after moving into supported housing.

To date, research has shown the importance of adopting normalization and SRV principles in service settings to facilitate the community integration of people with developmental disabilities. Only a small number of studies have examined the relationship between the presence of normalization and community integration for people with psychiatric disabilities. Moreover, these studies have used a narrow conceptualization of community integration, defined largely as physical presence in the community.

The current study builds on this previous research by examining the relationship of SRV in housing settings and different dimensions of community integration for people with psychiatric disabilities. The model guiding the study is presented in Figure 1. As shown in Figure 1, a greater presence of SRV in an individual's housing environment is hypothesized as being related to higher levels of physical integration, psychological integration, and psychological integration. In turn, higher levels of these different types of community integration are hypothesized as being related to a higher level of global life satisfaction.

**Figure 1:**
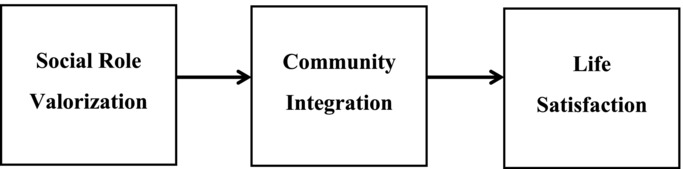
Study model showing hypothesized relationships between SRV, community integration, and life satisfaction.

## METHOD

### Participants

For the present study, a convenience sample was drawn from the client population of six community agencies delivering intensive case management services to people with psychiatric disabilities in a mid-size Canadian city. The sample was one of convenience in that the client's case manager initially informed them of the study, who in turn gave the contact information of those interested to the researchers. The participant pool was limited to approximately 289 clients across six programs (i.e., 17 case managers, each with an average of 17 clients per caseload). Of these 289 individuals, 73 participated, representing 25% of the client population.

The intensive case management programs of participants in the study are based on a combination of the strengths and psychosocial rehabilitation approaches. The case manager assists individuals to identify goals for service, develop an individual service plan, and meet the specified goals by providing support, counseling, advocacy, and linking to other resources. The service is designed to be portable, flexible and permanent, with the main goals of assisting individuals to become integrated into their community and to enjoy an improved quality of life.

### Measures

#### Global functioning

Participants’ current level of functioning as measured on the Global Assessment of Functioning (GAF) scale was used as a control variable in testing the proposed model. The GAF scale requires raters to consider an individual's psychological, social, and occupational functioning on a continuum of mental illness and mental health scored from 1 (suicidal acts, persistent danger of hurting self or others) to 100 (superior functioning; American Psychiatric Association, 2000). The GAF scale has been shown to be a reliable and valid measure for assessing the severity of psychiatric difficulties in a sample of people with severe mental illness (Jones, Thornicroft, Coffee, & Dunn, 1995). For purposes of the present study, the two interviewers who conducted the first interview made independent ratings for each participant, and an average GAF score for each participant was calculated. Inter-rater reliability for the two independent ratings was .79.

#### Social role valorization

For the purposes of this study, a shortened version of the PASSING rating system was used to evaluate participants’ housing environments in accordance with SRV principles. The shortened version of the PASSING system consisted of 26 items and was based on the three factor-based subscales developed by Flynn et al. (1999), namely, related to Program, Setting, and Accessibility.

The SRV Program subscale of the PASSING measure comprises 15 ratings that reflect the content (rather than structure) of a service program. A high rating on this subscale indicates that a program follows SRV principles by integrating program participants with nondisabled citizens, supporting participants’ age-appropriate choices and rights, facilitating acquisition by clients of personal possessions that enhance their image socially and improve their skills, encouraging positive and respectful interactions among clients, service staff, and members of the public, supporting participants’ uniqueness and individuality, focusing services on participants’ most pressing needs, and implementing the most effective and efficient interventions available.

The SRV Setting subscale comprises eight items that assess aspects of the physical setting in which a service is located. This subscale measures the extent the service setting blends into the surrounding neighborhood, the location is near other settings that are perceived positively in the context of appearance, use, and history, and the location facilitates community integration of its participants. The SRV Accessibility subscale comprises three items and measures the degree to which a program is conveniently accessible to its participants, their families, the public, and to a wide range of relevant community resources (e.g., banks, stores, restaurants, leisure facilities).

The subscale scores are calculated by summing the weighted scores of all the items comprising the factor (Wolfensberger & Thomas, 1983). The weighted score may also be expressed as a percentage of the total maximum weighted score possible on the subscale (Flynn et al., 1999).

In the absence of a standardized interview protocol to accompany the PASSING manual, semistructured client and staff interview protocols were created for use in the present study by a team of researchers trained in SRV principles and the use of PASSING. The interview protocol is based on the detailed criteria outlined in PASSING for the 26 items identified in Flynn et al.'s (1999) factor analyses. The clarity and conciseness of these interview protocols were established via pilot testing with three individuals who have a psychiatric disability and one case manager employed at one of the participating agencies.

In line with the original version of the PASSING, teams of two trained evaluators completed the ratings for each client, which were based on detailed criteria and guidelines outlined in the PASSING manual. Team members rated each of the 26 criteria independently on the basis of case manager and client interviews and observations of the service setting (i.e., the client's home). Following the field visit to the client's home and separate ratings by PASSING evaluators, a PASSING conciliation process was held. At this time team members discussed and resolved discrepancies in ratings and arrived at a consensus of how well the case management service scored on each of the 26 ratings.

Flynn and his colleagues (1999) found the subscales making up the short version of PASSING to have a high internal reliability. Specifically, the Cronbach's alpha was .92 for SRV Program, .85 for SRV Setting, and .79 for SRV Accessibility. Similarly, the Cronbach's alpha for the subscales in the current study were .87 for SRV Program, .86 for SRV Setting, and .78 for SRV Accessibility. Flynn, LaPointe, Wolfensberger, and Thomas (1991) calculated intra-class correlations computed on individuals raters’ preconciliation data for 633 PASSING assessments and found high levels of inter-rater reliability for the measure (at or above .90), as based on the average computed across raters in teams of five to nine members.

#### Physical integration

A condensed version of Segal and Aviram's (1978) external integration scale was used to measure physical integration. The scale was composed of 12 items assessing an individual's frequency of involvement in different activities outside their household in the past month, such as eating in a restaurant, visiting a library, and walking in a park. Possible responses varied from 0 (*never*) to 4 (*very often*). The potential total score on the scale ranged from 0 to 48 with higher scores representing relatively higher levels of physical integration. Cronbach's alpha for the measure in the present study was .70.

#### Psychological integration

The 12-item sense of community scale developed by Perkins, Florin, Rich, Wandersman, and Chavis (1990) was used to operationally define psychological integration. Items in the scale asked about sense of belonging, availability of help, feelings of influence, and emotional investment in relation to neighbors and the neighborhood. Respondents were asked to indicate if items were true (1) or false (0) in describing their beliefs and attitudes in these areas. The possible total score on the scale ranged from 0 to 12, with higher scores representing greater psychological integration in the neighborhood. Cronbach's alpha for the measure was .78.

#### Social integration

An expanded 13-item version of the scale developed by Aubry, Tefft, and Currie (1995) was used to measure social integration. Items in the scale asked respondents how often they had different kinds of social contact with neighbors ranging from fairly superficial (e.g., saying hello) to closer forms of contact (e.g., going out on a social outing). Response alternatives varied from 1 (never) to 5 (frequently). Potential total scores range from 13 to 65, with higher scores reflecting greater social integration. Cronbach's alpha for the scale was .92.

#### Life satisfaction

The five-item Satisfaction With Life Scale (SWLS; Diener, Emmons, Larsen & Griffin, 1985; Pavot & Diener, 1993) was used to assess an individual's global life satisfaction. The scale asks respondents to read five statements pertaining to life satisfaction and rate them on a scale ranging from 1 (*strongly disagree*) to 7 (*strongly agree*). Possible total score ranges from 5 to 35. Factor analyses have shown the scale to have a single dimension (Diener et al., 1985). The SWLS has been shown to have high internal reliability, with Cronbach alpha coefficients ranging from .79 to .89 in a variety of samples, and good test-retest reliability for temporal intervals up to 4 years (Pavot & Diener, 1993). Moreover, the SWLS has shown good convergent validity with other measures of subjective well-being and life satisfaction (Diener et al., 1985).

### Procedures

Case managers from participating programs informed their clients about the study. A member of the research team contacted interested clients (by telephone or via the case manager), who explained the purpose of the study and demands of participation. If clients agreed to participate, then two 90-minute interviews were scheduled.

Two interviewers conducted the first interview at the client's home to assess environmental variables pertinent to their housing according to the PASSING measure. The second interview, conducted at a place that was convenient and comfortable for the participant, was for the purpose of gaining information about their social networks, social support transactions, satisfaction with living arrangements, support received, and general life satisfaction. Participants in the study were paid $10 at the completion of each of the two interviews, for a total of $20.

The research team contacted a sample of 17 case managers of participating clients, who were also invited to participate in the study. A researcher conducted a 60-minute, semistructured interview using the PASSING tool to obtain additional information relevant to the client. In particular, case managers were questioned about the general nature of the case management services they provided, with particular emphasis on how these services were delivered relative to SRV principles.

The research team for the first set of client and staff interviews comprised eight students enrolled in a doctoral program in clinical psychology and two faculty members in psychology. Each member of the research team participated in a 5-day applied workshop on SRV principles and the PASSING tool, taught by an expert in the field of SRV. A second research team comprised two undergraduate and five graduate students in psychology and one faculty member. This team conducted the second phase of semistructured interviews regarding clients’ community integration and life satisfaction. Two individuals were members of both the first and second research teams. The study's methodology was approved by the research ethics board located at the researchers’ university.

## RESULTS

### Description of Sample

The sample of participants comprised slightly more men (53%) than women (47%). The average age of participants was 41 years old (standard deviation [*SD*] = 10.11, Range = 21–62 years), with the largest group (38%) falling in the 36–45-year-old category. A minority of the sample (37%) reported having completed high school. Almost three quarters of the sample (73%) identified social assistance as their primary source of income. The average income for the sample was $878.00 per month.

A majority of participants (71%) were living in independent living situations (i.e., private market apartments, house, or room). Other participants resided in supportive congregate housing (15%), board and care homes (11%), or supervised apartments (3%). The average length of time that participants had lived in their current housing was 3.8 years with a range of 1–48 years. A comparison of the study sample with the population of clients served by the participating agencies showed no significant differences in frequency distribution according to sex, age, or level of education. However, significant differences were found between the sample and population in the distribution of sources of income, with proportionally more individuals in the sample (12%) reporting employment as their primary source of income than in the client population (4%).

The most common self-reported primary psychiatric diagnosis of participants was schizophrenia (37%) followed by bipolar disorder (14%), affective disorder (10%), and schizoaffective disorder (10%). All participants, with the exception of three individuals, indicated that they had been hospitalized at least once in their lifetime for treatment of mental illness, with more than one-third (35%) reporting 10 or more hospitalizations.

### Statistical Analyses

Table 1 presents the descriptive statistics for the data on each of the variables examined in the analyses. To test the hypothesized relationships between SRV and community integration and between community integration and life satisfaction, a total of four multiple regression analyses were conducted using PASW 18 (SPSS 18). In particular, three multiple regressions were conducted to test the predicted relationships between the different measured aspects of SRV (i.e., program, setting, accessibility) and each of the community integration variables (i.e., physical integration, psychological integration, social integration). Subsequently, a fourth multiple regression analysis was conducted to test the predicted relationships of the three community integration variables to life satisfaction.

**Table 1:** Descriptive Information on Measurement Scales Used in the Analyses

Variable	N	M	SD	Potential range of scores	Obtained range of scores
Global Assessment of Functioning	73	53.50	11.66	0–100	27–80
SRV Program (*% )*	73	45.09	15.35	0–100	12–87
SRV Setting (%)	73	56.21	19.90	0–100	11–93
SRV Accessibility (%)	73	70.00	18.96	0–100	11–91
Physical Integration	67	14.52	6.59	0–48	2–28
Psychological Integration	67	7.28	3.01	0–12	0–12
Social Integration	67	25.87	10.83	13–65	14–60
Life Satisfaction	65	19.00	8.00	5–35	5–32

*Note*. m = mean; SD = standard deviation; SRV = social role valorization.

Prior to conducting the multiple regression analyses, missing data for a small number of cases (N = 6) were estimated using the regression estimation in the Missing Values Analysis of SPSS 18. Data for all of the variables were assessed for univariate outliers and the presence of skewness. No univariate outliers were found. The distribution for SRV Access was positively skewed and the distribution for social integration was negatively skewed. Consequently, square root transformations of these skewed variables were undertaken and multiple regressions were conducted with and without transformed variables. The same predictor variables were found to be significant in the multiple regressions, whether using transformed or nontransformed variables. Therefore, for ease of interpretability, the results from multiple regressions using nontransformed variables are reported in this article. The Mahanalobis distance for each case was calculated as part of each of the conducted multiple regressions to determine the presence of multivariate outliers. No multivariate outliers were found.

Table 2 presents a correlation matrix for the variables in the study. As shown in Table 2, bivariate correlations found a significant and positive relationship between sex of participants, global functioning, SRV, and community integration variables. As a result, sex of participants and global functioning were entered in the first step of hierarchical multiple regressions as control variables followed by the entry of predictor variables in subsequent steps. An examination of the multiple regressions found that sex was a nonsignificant predictor in all four regressions. Therefore, to maximize the power as well as produce the most interpretable results, sex of participants was removed as a control variable and multiple regressions were calculated with solely global functioning as a control variable.

**Table 2:** Correlation Matrix of Study Variables

	Variable	1	2	3	4	5	6	7	8	9
1	Sex	–								
2	Global Functioning	.29*	–							
3	SRV Program	.38***	.58***	–						
4	SRV Setting	.17	.11	.23*	–					
5	SRV Accessibility	−.19	−.15	−.12	−.02	–				
6	Physical Integration	.26*	.20	.34**	.17	.15	–			
7	Psychological Integration	.26*	.19	.38**	.21	−.01	.23	–		
8	Social Integration	.26*	.12	.33**	−.10	−.31*	.04	.29*	–	
9	Life Satisfaction	.23	.31*	.35**	.10	−.17	.32*	.37**	−.02	–

*Note*. Sex: Male (1), Female (2).

* p < .05.

** p < 0.01 level.

*** p < 0.001.

Table 3 presents the results of the hierarchical multiple regressions examining community integration variables as predictors after controlling for global functioning. In the first regression, the entry of SRV variables after controlling for global functioning of participants explained 11% of the variance in physical integration, *F change* (3, 68) = 2.99, p < .05. Among the SRV variables, a higher level SRV Program emerged as the sole significant predictor of a higher level of physical integration (*t* = 1.98, *sr^2^* = .05, *p* = .05).

**Table 3:** Hierarchical Multiple Regression Analyses Predicting Community Integration from Social Role Valorization in Housing Environments

	Type of community integration					
	
	Physical integration		Psychological integration		Social integration	
			
Predictor	ΔR^2^	β	ΔR^2^	β	ΔR^2^	β
Step 1	.05†		.03		.01	
Global Functioning		.22†		.18		.10
Step 2	.11*		.12*		.21**	
SRV Program		.31*		.35*		.43*
SRV Setting		.08		.15		−.24*
SRV Accessibility		.20†		.01		−.24*
Total *R^2^*	.16*		.15*		.22*	
*n*	73		73		73	

† p < .10.

* p < .05.

** p < .001.

In the second regression, testing SRV variables as predictors of psychological integration after controlling for global functioning of participants was significant, accounting for 12% of the variance in psychological integration, *F change* (3, 68) = 3.21, *p* < .05. Among the SRV variables, only a higher level of SRV Program emerged as a significant predictor of a higher level of psychological integration (*t* = 2.51, *sr^2^* = .08, *p* < .05).

In the third regression, the entry of SRV variables after controlling for global functioning of participants was also significant, explaining 21% of the variance in social integration, *F change* (3, 68) = 6.01, *p* = .001. All three SRV variables proved to be significant predictors of social integration in the regression. In particular, higher levels of SRV Program (*t* = 3.16, *sr^2^* = .12, *p* < .01), lower levels of SRV Setting (*t* = – 2.20, *sr^2^* = .06, *p* < .05), and lower levels of SRV Accessibility (*t* = – 2.24, *sr^2^* = .06, *p* < .05) predicted a higher level of social integration.

The final hierarchical multiple regression had three steps entering global functioning in the first step, followed by the entry of SRV variables in a second step, and community integration variables as predictors of life satisfaction in a third step (see Table 4). The entry of global functioning in the first step as control variable was significant, accounting for 11% of the variance in life satisfaction, *F change* (1, 71) = 8.53, *p* < .01. In this first step, higher levels of global functioning were related to higher levels of life satisfaction (*t* = 2.92, *sr^2^* = .12, *p* < .05). The entry of SRV variables in the second step was not significant. The entry of the community integration variables in the third step proved to be significant, explaining 18% of the variance, *F change* (3, 65) = 6.16, *p* = .001. Among the community integration variables, greater psychological integration (*t* = 3.09, *sr^2^* = .10, *p* < .01) and lesser social integration (*t* = – 2.17, *sr^2^* = .05, *p* < .05) were significant predictors of higher levels of life satisfaction. As well, greater physical integration (*t* = 1.95, *sr^2^* = .04, *p* = .06) approached significance as a predictor of higher levels of life satisfaction. Finally, SRV Accessibility emerged as a significant predictor in the third step (*t* = – 2.17, *sr^2^* = .05, *p* < .05), in the direction of lesser SRV Accessibility being associated with higher levels of life satisfaction.

**Table 4:** Hierarchical Multiple Regression Analyses Predicting Life Satisfaction From Social Role Valorization of Community Integration

	Life Satisfaction	
	
Predictor	ΔR^2^	β
Step 1	.11	
Global Functioning		.33*
Step 2	.06	
SRV Program		.24†
SRV Setting		.04
SRV Accessibility		−.13
Step 3^a^	.18	
Physical Integration		.21†
Psychological Integration		.34*
Social Integration		−.25†
Total *R^2^*	.35	
*n*	73	

a *Note*. SRV Accessibility showed a significant relationship in Step 3 with Life Satisfaction (*t* = − 2.17, *sr^2^* = .05, *p* < .05).

† p < .10.

* p < .05.

## DISCUSSION

The main goal of the study was to examine the relationship of SRV to community integration and life satisfaction in a sample of people with psychiatric disabilities living in the community. The findings suggest that the promotion of SRV values through the functional features of housing environments and their associated services is facilitative of community integration for people with psychiatric disabilities. In particular, housing environments that support client individualization, autonomy, and rights, facilitate the development of abilities to live independently, and project an image of residents who are similar to and integrated with people living in proximity are positively related to the different types of community integration. Overall, the findings indicate that the relationship between SRV Program and life satisfaction is mediated by psychological integration. In particular, higher levels of SRV Program are predictive of higher levels of psychological integration. In turn, higher levels of psychological integration are predictive of higher levels of life satisfaction.

These findings are consistent with previous research on normalization that demonstrated its relationship to adaptive functioning and participation in community activities and with community residents (Hull & Thompson, 1981a, 1981b, 1984). This study extends previous research by examining housing environments that include, in large part, supported housing programs in which individuals with psychiatric disabilities are assisted to live in regular housing in different parts of the city (Rog, 2004; Tabol, Drebing, & Rosenheck, 2010). As well, the study goes beyond previous research by using the PASSING tool, which is based on SRV theory, and by examining community integration in a more comprehensive manner than simply focusing on adaptive functioning and presence in the community.

The level of SRV reflected on the three PASSING subscales in our study exceeds the average level achieved on these measures, for 633 programs located in the United States, Canada, Australia, and the United Kingdom as reported by Flynn et al. (1999). In particular, the average SRV Program score in our study was 45.09 (*SD* = 15.35) compared to an average of 21.10 (*SD* = 17.30) calculated for the programs examined by Flynn et al. (1999). The average SRV Setting (*M* = 47.50, *SD* = 24.10) and SRV Accessibility (*M* = 55.20, *SD* = 27.60) in the Flynn et al. study are also lower than the average SRV Setting (*M* = 56.21, *SD* = 19.90) and SRV Accessibility (*M* = 70.00, *SD* = 18.96) in our study.

These differences in SRV scores may be explained by several factors. The fact that Flynn and his colleagues (1999) examined a wide range of types of programs delivered for wide range of populations may explain, at least in part, these differences. Also, a majority of participants in our study resided in supported housing. This approach, which supports individuals to live in regular housing, shares many similar values to those of SRV (Flynn & Aubry, 1999; Tabol et al., 2010). At the same time, the average score on the SRV Program subscale for the housing settings we assessed is still less than half the possible total score, suggesting that even in supported housing contexts, the environment falls short of the ideal in facilitating social role valorization.

Our findings highlight the importance of the functional aspects of housing environments and services as scores on the SRV Program subscale were significantly and positively related to all three types of community integration examined in the study. High scores on this subscale reflect environments and services that integrate participants with nondisabled citizens, match type of support with participant needs, promote age-appropriate and culture-appropriate personal appearance and activities, interact with participants in a respectful and positive manner, facilitate the development of abilities, promote autonomy and personal rights, and expose them to age-appropriate challenges (Flynn et al., 1999).

Given the attributes of housing environments and services that are rated on the SRV Program subscale, it is understandable that they would facilitate physical presence in the community, social contact with neighbors, and ultimately a sense of belonging in the neighborhood. These attributes share many similarities with recovery-oriented services that are also expected to promote community integration (Davidson et al., 2007; Farkas et al., 2005; Jacobson & Curtis, 2000; Sowers, 2005).

Levels of SRV Accessibility and SRV Setting were associated with levels of social integration but in the direction opposite to what we hypothesized. In particular, lower levels of SRV Accessibility and SRV Setting were related to higher levels of social integration. A possible explanation for the negative relationship between SRV Accessibility and social integration is that high levels of SRV Accessibility mean that participants have easy access to community resources such as banks, stores, and restaurants because of the proximity to their housing. However, the location may also possibly limit participant's opportunities to have contact with neighbors because of the nature of the neighborhoods in which they live. In particular, neighborhoods facilitating ease of access to community resources can be expected to have a mixed make-up (i.e., commercial and residential), with a smaller number of residents living there. As a result, these neighborhoods may be less conducive to social integration.

The relationship between higher levels of SRV Setting and lower levels of social integration may be the result of the social isolation that is often experienced by people with psychiatric disabilities living in regular housing. Higher levels of SRV Setting are in line with housing that is well assimilated and congruent with other housing in a regular type of neighborhood.

In these situations, there may actually be fewer opportunities for people with psychiatric disabilities to interact with neighbors. There is evidence that tenants in supported housing programs living in regular housing report being socially isolated (Tsai et al., 2012). The average level of social contact reported by our participants is actually quite low, with the average response on individual items corresponding to “rarely” having different types of contact with neighbors. This low level of contact is similar to previous findings of research examining the social contact of people with psychiatric disabilities with their neighbors (Aubry & Myner, 1996; Yanos et al., 2012). Ultimately, living in regular housing in residential neighborhoods may actually work against having contact with nondisabled neighbors.

Among the different types of community integration, higher levels of psychological integration emerge as being significantly related to higher levels of life satisfaction. Previous research examining the relationship between different types of community integration and life satisfaction among people with psychiatric disabilities reported a similar result, with only psychological integration having a significant positive association with life satisfaction (Aubry & Myner, 1996). Other research investigating sense of community in the general population also found it to have a positive relationship with life satisfaction, general well-being, and mental health functioning (Davidson & Cotter, 1991; Farrell, Aubry, & Coulombe, 2004; Prezza, Amici, Roberti, & Tedeschi, 2001). In addition, the relationship between physical integration and life satisfaction approached statistical significance in our study. It makes sense that higher levels of physical activities in the community contribute to greater life satisfaction.

The finding that higher levels of social integration are related to lower levels of life satisfaction is unexpected, particularly in light of the positive and significant correlation between psychological integration and social integration in our study. As well, previous research has found a positive relationship between social integration and life satisfaction in the general population (Farrell et al., 2004; Prezza et al., 2001). In the case of the current study, the findings suggest that higher levels of social contact with neighbors are more likely to contribute to difficulties rather than yield benefits that contribute to life satisfaction.

Among SRV variables, a higher level of accessibility associated with a participant's housing environment was associated with lower levels of life satisfaction. It is possible that the characteristics of neighborhooods that afford greater accessibility to community resources may also contribute to lower levels of life satisfaction. In particular, we expect housing to have high levels of accessibility in inner-city neighborhoods, where there is often a preponderance of crime and poverty (Curley, 2005).

### Limitations

Our study has several limitations that need to be taken into account when interpreting the findings. The first limitation is the relatively small sample size, which restricts the power to detect significant relationships. A second limitation involved the use of a convenience sampling method in which case managers identified for the researchers potential participants. At the same time, participants were representative of the consumer population on most of the demographic characteristics on which we had population data.

A third limitation is the exclusive focus on perceptions of the neighborhood in operationalizing psychological integration and on social contact with neighbors in defining social integration. Wong and Solomon (2002) argue for a broadening of social integration to include a focus on social networks and the exchange of social support. A fourth limitation–the measure used to assess social integration–may have been interpreted in an ambiguous manner by people living in congregate housing who have their own room or even apartment but share common areas. In these cases, participants may have defined their house mates as neighbors, complicating the interpretation of the findings from this measure.

Despite these limitations, the current study highlights the importance of the presence of SRV in housing environments for people with psychiatric disabilities. In particular, our study demonstrates the importance of housing situations that afford its residents opportunities to assume valued social roles in the community and in relationships with valued nondisabled individuals, thereby serving to facilitate their community integration. From a policy perspective, our findings linking SRV to community integration argue for the development of supported housing program that supports individuals to live in regular housing as the preferred approach to combining housing and support for people with psychiatric disabilities.

Wong, Filoromo, and Tennille (2007) identified four core principles behind supported housing: (a) housing is a basic right for people with psychiatric disabilities, (b) people with psychiatric disabilities are to live in housing as regular tenants and community members, (c) empowerment is the practice goal for the relationship between consumers and support staff, and (d) housing and mental health support are functionally separate. These core principles correspond well to the goal and tenets of SRV. A major challenge faced by mental health systems is how to transform housing and service environments in this direction.
